# Molecular identification of bronchopulmonary neuroendocrine tumours and neuroendocrine genotype in lung neoplasia using the NETest liquid biopsy

**DOI:** 10.1093/ejcts/ezaa018

**Published:** 2020-02-11

**Authors:** Pier Luigi Filosso, Kjell Öberg, Anna Malczewska, Anna Lewczuk, Matteo Roffinella, Harry Aslanian, Lisa Bodei

**Affiliations:** 1 Department of Surgery, University of Torino, Torino, Italy; 2 Department of Endocrine Oncology, University Hospital, Uppsala, Sweden; 3 Department of Endocrinology, Medical University of Silesia, Katowice, Poland; 4 Department of Medicine, Endocrinology Unit, Medical University of Gdansk, Gdansk, Poland; 5 Department of Medicine, Yale University School of Medicine, New Haven, CT, USA; 6 Department of Radiology, Memorial Sloan Kettering Cancer Center, New York, NY, USA

**Keywords:** Bronchopulmonary carcinoid, Neuroendocrine, Carcinoid, NETest, Lung surgery, Lung cancer, Chromogranin A

## Abstract

**OBJECTIVES:**

Diagnosing lung neuroendocrine neoplasia (NEN) requires a biopsy or an operation. We evaluated a ‘liquid biopsy’ (NETest) as an *in vitro* diagnostic tool for identifying NEN and compared it to chromogranin A (CgA).

**METHODS:**

We identified 4 study cohorts: patients with bronchopulmonary carcinoids (*n *=* *99, including 62 typical and 37 atypical carcinoids), lung cancers [*n *=* *101, including 41 adenocarcinomas, 37 squamous carcinomas (SQC), 16 small-cell lung cancers and 7 large-cell neuroendocrine carcinomas]; benign disease (50 idiopathic pulmonary fibrosis) and healthy controls (*n *=* *102). Transcript levels measured quantitatively (activity scores: 0–100) were compared to CgA (enzyme-linked immunosorbent assay; normal < 109 ng/ml) levels.

**RESULTS:**

The results of the NETest were positive (>20) in 94% of patients with bronchopulmonary carcinoid compared to 8% of the controls (Fisher’s exact test; *P *<* *0.001) and were significantly more accurate as a diagnostic test (McNemar’s test; *P *<* *0.001, *χ*^2^* *=* *72) than was CgA (positive: 19% bronchopulmonary carcinoid, 15% controls). Small-cell lung cancers (87%), large-cell neuroendocrine carcinomas (86%), adenocarcinoma (42%) and SQC (35%) were also NETest-positive. Increasing the NETest cut-off score to >40 was useful for detecting all NENs and differentiating these tumours from either controls/benign lung diseases (specificity 97%) or adenocarcinoma/SQC (specificity 94%). CgA was positive in 15–44% irrespective of pathology and had no diagnostic value.

**CONCLUSIONS:**

A gene-based liquid biopsy is an effective and accurate method for diagnosing lung tumours with neuroendocrine gene expression. CgA has no value. An NETest score >40 provides an accurate (94–97%) rule-in for the diagnosis of NEN and a rule-out for benign and other neoplastic diseases. Because neuroendocrine gene expression is associated with a poor prognosis, NETest levels may have utility both in the diagnosis of and the treatment stratification for lung neoplasia.

**Figure:**
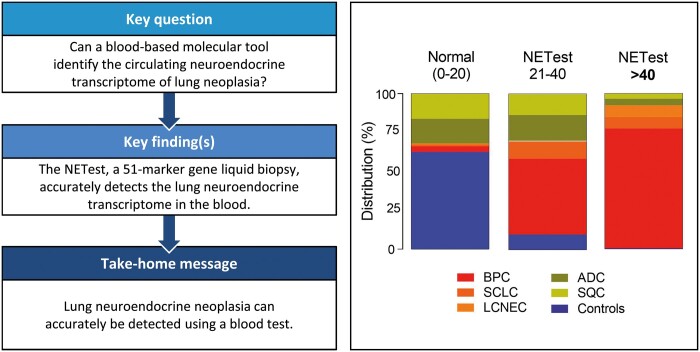

## INTRODUCTION

Lung neoplasia is usually a serendipitous diagnosis or is based on diverse, often covert, clinical symptoms. Tumours are rarely identified early because screening programmes fail to detect lesions until they have reached an appreciable size [1]. Although specific symptoms such as coughing, haemoptysis, bronchospasm, pain and shortness of breath may be harbingers, they usually indicate late disease. Non-specific symptoms like fatigue, loss of weight, neural manifestations and anaemia suggest an even more dire prognosis. A minority (3–5%) present with hormonally related symptoms such as carcinoid syndrome, Cushing’s disease, acromegaly or inappropriate antidiuretic hormone secretion, suggesting a neuroendocrine phenotype [2].

A key limitation in diagnosis is that no effective blood biomarkers exist. Image identification of a lung tumour rarely characterizes the specific neoplastic type, and bronchoscopy or needle biopsy and a histological/cytological diagnosis are required to define the type. These approaches are invasive, and tissue samples obtained are not only random but limited by tumour heterogeneity [3]. Strategies that identify and objectively measure the molecular genomic characteristics of a tumour as opposed to a descriptive histological analysis show promise [4]. Considerable information now exists to support the idea that blood sampling can provide useful oncological information. A circulating neoplastic molecular signature could limit invasive biopsies, define therapeutic targets and provide a real-time monitoring tool to evaluate disease status. Such strategies, or ‘liquid biopsies’, already have value in lung neoplasia, e.g. for monitoring treatment responses to epidermal growth factor receptor (EGFR) inhibitors through identification of mutation T790M in circulating tumour DNA [5], and are becoming standards of care [6, 7].

Lung cancers that express neuroendocrine phenotypes include neuroendocrine neoplasias (NENs) such as ‘carcinoids’ or neuroendocrine tumours (NETs), small-cell lung cancers (SCLC) and large-cell neuroendocrine carcinomas (LCNECs) as well as some non-small-cell lung cancers (NSCLC) [2]. These represent ∼25% of all lung neoplasias. There is currently no well-established liquid biopsy that can detect neuroendocrine differentiation.

Recently, a multianalyte molecular assay (51 transcripts, NETest) was developed to identify NET disease in the blood [8, 9]. Recent reports confirm that NETest genes are evident in lung neuroendocrine transcriptomes [10] and can be identified in neuroendocrine lung cell lines [10]. The test has utility as a diagnostic [11] tool and as a monitor for surgical efficacy in lung NETs [12]. NETest gene expression has also been independently identified in tumour tissue from lung adenocarcinomas (ADCs) and lung LCNECs [13]. Up to 31% of all lung ADCs highly express NETest genes irrespective of histological status [13]. Samples with high expression exhibit the poorest prognosis of all tumours evaluated [13]. The utility of the NETest to detect neuroendocrine signatures in lung diseases remains to be determined.

We evaluated the diagnostic utility of the assay in blood for different lung cancers and compared its accuracy to that of chromogranin A (CgA). Different cut-off levels of the NETest scores were assessed to identify the likelihood of the lung lesion being neuroendocrine. The latter is of particular relevance because expression of NETest genes in tumour tissues is associated with an adverse prognosis [13].

## SUBJECTS AND METHODS

### Patients

Patients provided informed consent for the blood measurements (HIC0805003870, 15 June 2017). Whole blood [5 ml; messenger RNA (mRNA)] and plasma (1 ml; CgA) were collected.

### Diagnostic cohort (*n* = 303)

The participants in this multicentre, prospective cohort were collected between June 2017 and December 2018. The cohort included patients and non-affected family members attending oncology, endocrinology and pulmonology out-patient clinics and therefore represented a real-world evaluation of subjects (Table 1). Inclusion criteria included histological confirmation of disease. No exclusion criteria were used. Controls (*n *=* *102) were asymptomatic and considered to be in good health. The bronchopulmonary carcinoid (BPC) cohort included 99 subjects [62 with typical carcinoids (TCs) and 37 with atypical carcinoids (AC)]. Other lung neoplasias included 23 other neuroendocrine neoplasms (LCNEC: *n* = 7; SCLC: *n* = 16), ADC (ADC: *n* = 41) and squamous cell carcinoma (SQC: *n* = 37) (Table 1). Subjects with idiopathic pulmonary fibrosis (IPF) (*n* = 50) were also collected. Fifty-two percent of patients with carcinoids were stable at the blood draw; 47 (48%) had progressive disease. The majority of patients with ADC (66%) and SQC (81%) had disseminated disease.

**Table 1:** Patient demographics

Variables	Controls (*n *=* *102)	IPF (*n *=* *50)	BP carcinoids (*n *=* *99)	ADC (*n *=* *41)	SQC (*n *=* *37)	LCNEC (*n *=* *7)	SCLC (*n *=* *16)
Age (years), median (range)	49 (22–89)	65 (32–85)	59 (20–82)	64 (36–79)	65 (41–82)	70 (56–77)	64 (32–79)
Gender (male:female)	29:73	35:15	36:63	26:15	24:13	4:3	10:6
Tumour histological type/T stage (TNM classification for lung tumours), *n* %	N/A	N/A	TC: 62 (62) T1: 50 (80) T2: 8 (13) T3: 1 (2) T4: 3 (5) AC: 37 (37) T1: 9 (24) T2: 12 (32) T3: 12 (32) T4: 4 (12)	T1: 10 (24) T2: 16 (39) T3: 7 (17) T4: 8 (20)	T1: 2 (5) T2: 7 (19) T3: 12 (32) T4: 16 (44)	T1: 1 (14) T2: 6 (86) T3: 0 (0) T4: 0 (0)	T1: 0 (0) T2: 1 (6) T3: 8 (50) T4: 7 (44)
Disease extent (metastases status), *n* %	N/A	N/A	Advanced metastatic disease (distant metastases): 29 (29) TC-M1: 12 (20) AC-M1: 17 (46)	Advanced metastatic disease (distant metastases): 27 (66)	Advanced metastatic disease (distant metastases): 31 (84)	Advanced metastatic disease (distant metastases): 3 (43)	Advanced metastatic disease (distant metastases): 13 (81)
Disease status (RECIST 1.1), *n* %	N/A	N/A	SD: 52 (52) PD: 47 (47)	SD: 14 (34) PD: 27 (66)	SD: 14 (38) PD: 23 (62)	SD: 2 (29) PD: 5 (61)	SD: 6 (37) PD: 10 (63)
Current treatment, *n* %	N/A	N/A	Presurgery: 31 (31) PRRT: 3 (3) SSA: 22 (22) Currently not treated: 43 (43)	Presurgery: 13 (32) Prechemotherapy: 8 (20) Chemotherapy:^a^ 14 (34) Currently not treated: 6 (14)	Presurgery: 2 (5) Prechemotherapy: 5 (13) Chemotherapy:^a^ 16 (44) Radiation: 4 (11) Currently not treated: 10 (27)	Presurgery: 5 (61) CapTEM: 2 (29)	Chemotherapy: 10 (63)^a^ Currently not treated: 6 (37)

a Cisplatinum-based therapy (with or without vinorelbine or etoposide).

AC: atypical carcinoid; ADC: adenocarcinoma; BP: bronchopulmonary; CapTEM: capecitabine and temozolomide; IPF: idiopathic pulmonary fibrosis; LCNEC: large-cell neuroendocrine carcinoma; N/A: not applicable; PD: progressive disease; PRRT: peptide receptor radionuclide therapy; RECIST: Response Evaluation Criteria in Solid Tumors; SQC: squamous cell carcinoma; SCLC: small-cell lung cancer; SD: stable disease; SSA: somatostatin analogue; TC: typical carcinoid; TNM: tumour/node/metastasis.

### Biochemical assays

Five millilitre of whole blood was collected in K2 ethylenediaminetetraacetic acid tubes and snap frozen. Plasma CgA samples were collected at the same time (Plasma Preparation Tubes). Tubes were anonymously coded and stored at −80°C within 2 h of collection. Randomly selected coded blood samples were sent de-identified to Wren Laboratories, Branford, CT, USA.

#### NETest

A 2-step protocol [ribonucleic acid (RNA) isolation, complementary DNA, polymerase chain reaction] was followed in a clinically certified laboratory (Wren Laboratories CL-0704, CLIA 07D2081388) [8, 12] in the USA. Transcripts (messenger RNA; Supplementary Material, Table S1) were isolated from ethylenediaminetetraacetic acid-collected whole blood samples (blood mini kit, Qiagen, Valencia, CA, USA) and a real-time polymerase chain reaction was performed. Target levels were normalized and quantified. Final results are expressed as an activity index (NETest score) from 0 to 100 [12]. The normal cut-off is 20. NETest values ≤40 are considered representative of ‘stable’ disease and are thus categorized; values 41–100 reflect ‘progressive’ disease based on imaging (Response Evaluation Criteria in Solid Tumours) changes.

#### Chromogranin A enzyme-linked immunosorbent assay

CgA was measured using NEOLISA™ CgA kits (Euro Diagnostics, Malmo, Sweden). A cut-off of 108 ng/ml defined the upper limit of normal [12].

### Statistical analysis

Intergroup analyses were undertaken using 2-tailed non-parametric tests [Kruskal–Wallis (KW) (with Dunn’s correction) for multiple samples; Mann–Whitney *U*-test for 2 groups, e.g. AC versus TC; Fisher’s exact test for proportions or McNemar’s test for matched NETest/CgA results]. Receiver operator curve analysis defined the diagnostic accuracy of each biomarker [14]. An area under the curve (AUC) value >0.9 is considered an excellent diagnostic tool; values 0.8–0.9 are considered good, whereas values <0.8 are considered fair to poor [14]. The diagnostic odds ratio (DOR), the positive likelihood ratio (PLR) and the negative likelihood ratio (NLR) were calculated. DORs range from zero to infinity; higher values indicate a better discriminative performance. A value of 1 indicates that a test has no discriminant ability. The PLR is used to identify a ‘rule-in’ test. Good diagnostic tests typically exhibit a PLR >10. The NLR provides information to ‘rule-out’ the usefulness of a test. Values <0.1 indicate a useful test. Prism 6.0 for Windows (GraphPad Software, La Jolla, CA, USA; www.graphpad.com) and MedCalc Statistical Software version 16.2.1 (MedCalc Software bvba, Ostend, Belgium; http://www.medcalc.org; 2017) were utilized. Data are presented as mean (standard deviation) and median (interquartile ranges).

## RESULTS

### The NETest as an *in vitro* diagnostic tool: lung diseases versus controls

**Figure 1:**
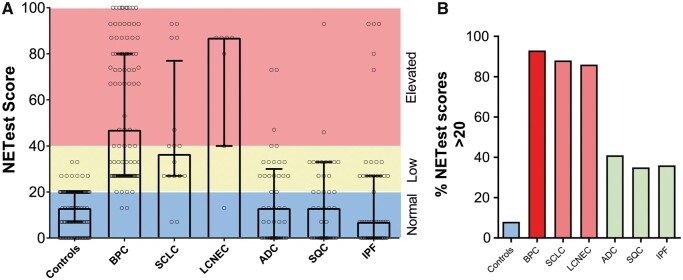
NETest scores in controls and in patients with lung neoplasia and benign disease. (**A**) NETest scores stratified by normal (0–20) (blue), low (21–40) (green) and elevated (>40) (red) levels in each of the cohorts. The Kruskal–Wallis statistic was 154 (*P *<* *0.001). (**B**) The percentage of positive NETest scores (21–100) in individual cohorts. In lung disease, the percentage of subjects with positive scores ranged from 35% to 93%. Neuroendocrine genotype tumours (red); control subjects (blue); non-small-cell lung cancer. ADC: adenocarcinoma; BPC: bronchopulmonary carcinoids; IPF: idiopathic pulmonary fibrosis (green); LCNEC: large-cell neuroendocrine carcinoma; SCLC: small-cell lung cancer; SQC: squamous cell carcinoma.

The results of the NETest were positive in 8 (8%) controls [13.1 (9) 13 (7–20)] (Fig. 1A and B). In comparison, it was positive in 93 (94%) patients with BPC [56 (29) 47 (27–80); *P *<* *0.001, KW with Dunn’s correction] and exhibited no differences in patients with AC [62 (30) 67 (27–90)] and those with TC [52 (28) 36.5 (27–80), *P *=* *0.15; Mann–Whitney]. The NETest was positive in 14 (88%) patients with SCLC [44 (29) 37 (27–77), *P *<* *0.001; KW] and in 86% of patients with LCNEC [69 (30) 87 (40–87), *P *<* *0.001; KW]. It was also positive in 17 (42%) patients with ADC [19 (19) 13 (0–30), *P *=* *0.87; KW] and in 13 (35%) patients with SQC [18 (19) 13 (0–33), *P *>* *0.99; KW]. IPF also exhibited positive scores in 18 patients (36%) [18 (26) 7 (0–27), *P *>* *0.99; KW].

The diagnostic metrics are included in Supplementary Material, Table S2. The highest sensitivity was noted for BPC (94%) but was also elevated for SCLC (88%) and LCNEC (86%). Sensitivities <50% were noted for ADC (41%), SQC (35%) and IPF (36%). The highest AUC values were those of BPC (0.93), SCLC (0.9) and LCNEC (0.89) (all *P *<* *0.001; Supplementary Material, Fig. S1A), which identified the NETest as an excellent biomarker for identifying tumours with a neuroendocrine phenotype. The NETest was not effective for differentiating between BPC and poorly differentiated NETs (SCLC and LCNEC, AUC: 0.52), confirming that it functioned similarly in both types of lung NENs. The NETest was less effective for differentiating controls from ADC (0.67), SQC (0.64) and IPF (0.64), which was consistent with its lower value as a diagnostic tool for these tumours or a benign condition.

The highest PLRs were identified for BPC (12), SCLC (11.2) and 10.9 (LCNEC) with lower values for ADC (5.3), SQC (4.5) and IPF (4.6). These findings indicate that the NETest is a useful tool to confirm the diagnosis of neuroendocrine lung neoplasia. The lowest NLR were identified for BPC (0.07), SCLC (0.14) and LCNEC (0.16), demonstrating utility as a rule-out biomarker for lung NENs.

Evaluation of the DOR identified that its level was elevated and indicative of an effective diagnostic biomarker for BPC (182, *P *<* *0.001), SCLC (82, *P *<* *0.001) and LCNEC (71, *P *<* *0.001). Levels in ADC (8.3), SQC (6.3) and IPF (6.6), although significant (*P *<* *0.003), were 10-fold less, indicative of demonstrably lower diagnostic utility for these tumour types.

### Chromogranin A as an *in vitro* diagnostic tool: lung diseases versus controls

**Figure 2:**
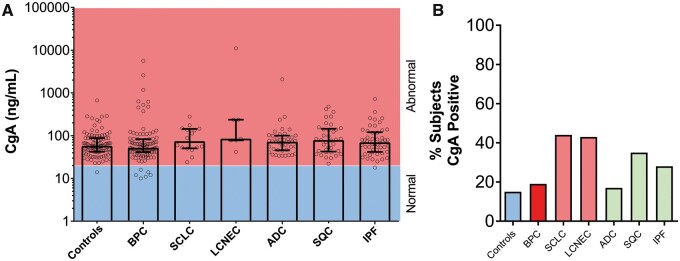
Chromogranin A (CgA) levels in controls and in patients with benign disease and lung neoplasia. (**A**) CgA stratified by normal (blue) or abnormal (red) in each of the cohorts. No significant differences were evident. The Kruskal–Wallis statistic was 13.6 (*P* > 0.05). (**B**) Percentage of CgA positive responses in the different cohorts. In lung disease, a positive CgA response (>108 ng/ml) ranged from 17% to 44%. Neuroendocrine genotype tumours (red); control subjects (blue); non-small-cell lung cancer. ADC: adenocarcinoma; BPC: bronchopulmonary carcinoids; IPF: idiopathic pulmonary fibrosis (green); LCNEC: large-cell neuroendocrine carcinoma; SCLC: small-cell lung cancer; SQC: squamous cell carcinoma.

CgA levels were was positive in 15 (15%) controls [80 (81) ng/ml 58 (42–88)] (Fig. 2A and B) and in 19 (19%) patients with BPC [178 (628) 52 (41–83), *P *>* *0.99, KW]. No differences were identified between AC [95 (207) 48 (36.5–63.5)] and TC [227 (776) 62 (41–109); *P *=* *0.06; Mann–Whitney]. CgA levels were positive in 7 (44%) SCLC [100 (68) 74 (51–144), *P *=* *0.97; KW] and in 43% of patients with LCNEC [1680 (4113) 85 (78–237); *P *=* *0.17; KW]. It was also positive in 7 (17%) patients with ADC [128 (316) 72 (46–101), *P *>* *0.99; KW] and in 13 (35%) patients with SQC [119 (112) 79 (43,145), *P *=* *0.46; KW]. CgA levels in patients with IPF were positive in 14 (28%) [103 (114) 70 (42–121), *P *>* *0.99, KW].

Diagnostic metrics are shown in Supplementary Material, Table S3. Sensitivity for SCLC and SQC was 44% and 35%, respectively. The AUC for SCLC and SQC was 0.65 and 0.6, respectively (Supplementary Material, Fig. S1B). The PLR for SCLC was 3.0, but the NLRs for all others were ∼1 (range 0.66–0.97). The DOR was highest for SCLC (4.5; *P *<* *0.009) and SQC (3.14; *P *<* *0.01). Although the AUC for BPC versus that for the SCLC and LCNEC was 0.68, a low proportion of patients in each cohort (BPC: 19%, SCLC + LCNEC: 43%) were positive, making diagnostic utility low. Overall, CgA was not an effective biomarker for any lung disease evaluated.

### Comparisons between the NETest and chromogranin A as an *in vitro* diagnostic test

Matched whole blood: plasma samples were available from all patients. The NETest was significantly better than CgA for diagnosing BPC (Supplementary Material, Table S4; McNemar’s test; *P *<* *0.001; *χ*^2^* *=* *72). A significant difference was also identified for SCLC (*P *=* *0.045; *χ*^2^* *=* *4.0) whereas ADC was marginal (*P *=* *0.055; *χ*^2^* *=* *3.7). In the BPC cohort, the NETest provided added value as a diagnostic in 74/99 (75%). In the 99 image-positive BPCs, 19 were both NETest-positive and CgA-positive whereas 74 were NETest-positive and CgA-negative. The NETest correlated with disease detection and identified BPC when CgA levels were normal. The calculated added value of an NETest as a diagnostic tool for BPC versus that of CgA was 390% (74/19). For SCLC, the NETest identified an additional 8 patients who were CgA-negative—providing an added value of 8/6 (133%). For LCNEC, the NETest identified an additional 4 patients who were CgA-negative—providing an added value of 4/2 (200%). Overall, both well-differentiated and poorly differentiated NETs were more effectively diagnosed by the NETest (added value 86/27 = 319%) than by CgA. One hundred and thirteen (92.6%) patients with BPC/SCLC/LCNECs were NETest-positive. Seven were NETest/CgA-negative and 2 were CgA-positive. Combining NETest and CgA did not improve the diagnostic value (113 vs 115, Fisher’s exact test; *P *=* *0.80; Supplementary Material, Table S5).

### Evaluation of the NETest as a screening tool

**Figure 3:**
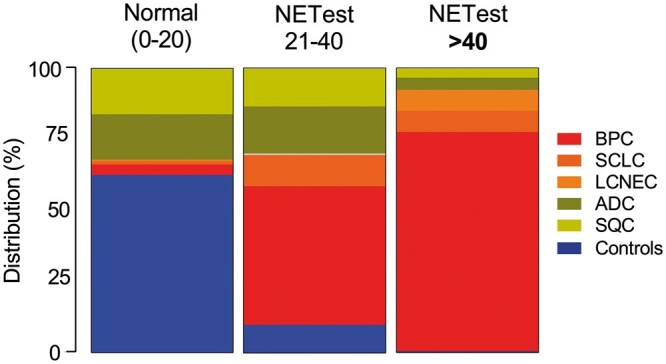
Distribution of NETest scores for controls and individual tumour types. An elevated score (>40) was predominantly identified in BPC and was consistent with a progressive neuroendocrine disease phenotype. ADC: adenocarcinoma; BPC: bronchopulmonary carcinoids; LCNEC: large-cell neuroendocrine carcinoma; SCLC: small-cell lung cancer; SQC: squamous cell carcinoma.

Although the NETest results were positive in a high proportion of patients with lung NENs (86–88%), they were also positive in some patients with ADC and SQC (Fig. 3). However, more patients with BPC were positive than those with ADC (*P *<* *0.001; Fisher’s exact test) or with SQC (*P *<* *0.001; Fisher’s exact test). More patients with SCLC were positive than those with ADC (*P *<* *0.003; Fisher’s exact test) or with SQC (*P *<* *0.001; Fisher’s exact test) and also LCNEC versus ADC (*P *=* *0.023; Fisher’s exact test) or SQC (*P *=* *0.015; Fisher’s exact test). The NETest therefore detected neuroendocrine lung disease. Nevertheless, 35–42% of patients with ADC and SQC also exhibited a circulating neuroendocrine signature. This finding is consistent with those from recent tissue analyses [13] and suggests that the NETest also identifies a subset of ‘non-NENs’ that will have a neuroendocrine genotype and likely a poor prognosis.

To investigate this concept, we evaluated a higher cut-off value of 40 as a discriminant. The rationale is that we previously identified that NETest scores >40 effectively identified progressive lung NEN disease [15, 16]. We hypothesized that stratification using an NETest score of 40 might identify ‘aggressive’ disease and better differentiate BPC from ADC and SQC.

An elevated NETest score (>40) was evident in 51 (52%) patients with BPC compared to 5 (31%) with SCLC (*P *=* *0.17; Fisher’s exact test) and 5 (71%) LCNEC (*P *=* *0.44; Fisher’s exact test). Of the patients with BPC with NETest score >40, 43 (84%) exhibited progressive disease consistent with identification of an aggressive phenotype. Three (7%) patients with ADC and 2 (5%) with SQC also demonstrated an elevated NETest score. We presume that these 5 tumour samples may exhibit high levels of neuroendocrine gene transcription as has been noted in a large National Institutes of Health-funded Cancer Genome Atlas study of NSCLC [13]. An elevated NETest score >40 is therefore highly significantly associated with aggressive BPC (Fisher’s exact test: *P *<* *0.001 versus ADC and SQC; Supplementary Material, Table S6) and differentiating lung NEN from controls/IPF (Supplementary Material, Table S7). The specificity versus ADC (93%) and SQC (95%) was elevated as was that for PLR (7–9.5) and DOR (12.9–17.9, *P *<* *0.001).

## DISCUSSION

A critical issue in the diagnosis and management of lung NEN is the lack of an effective blood biomarker. Moreover, there is currently no well-established liquid biopsy that can detect neuroendocrine transcriptome activation, which is identifiable in one-third of cases of NSCLC, irrespective of histological diagnosis [13]. We examined the diagnostic utility of an NET-specific gene assay to detect NEN and circulating NET transcripts in different lung cancers.

Currently, molecular tools are being evaluated to better define the landscape of lung tumours [17, 18]. Existing classifications, e.g. TC or AC, will likely be supplanted by other pathological or molecular taxonomies. These range from recent histopathological groupings, including early aggressive primary high-grade NETs versus secondary high-grade NETs and indolent NET [17] to neuroendocrine molecular signatures detectable in tissue and blood [13]. It remains challenging, however, to accurately predict an individual tumour’s behaviour because sophisticated tissue-based molecular tools are unfortunately not available [19]. Given the need for strategies to easily define lung neoplasia at the molecular level, we investigated the use of a blood-based multianalyte molecular tool.

The NETest accurately (93%) differentiated BPCs from controls when a cut-off of 20 was used whereas CgA detected only 19%. Combining CgA with the NETest did not improve the detection rate. Given that CgA is normal in ∼60% of lung carcinoids [20], it is not surprising that it performed poorly. Similarly, the NETest accurately identified SCLC (87%) and LCNEC (86%). Both these entities have well-described neuroendocrine phenotype/genotypes, and the NETest results are therefore consistent with the identification in the blood of the NET gene signature [15]. One clinical use of the NETest in these patients is facilitating an earlier diagnosis (Supplementary Material, Fig. S2). SCLC comprises ∼20% of all lung neoplasias and has the lowest survival rate of any lung cancer. This result reflects not only their aggressiveness but also that the majority of patients are diagnosed with advanced disease [21]. Furthermore, treatment, typically chemotherapy, is monitored using neuron-specific enolase, which is non-specific, is not consistently detected in these tumours and is not considered to meet the criteria of the US National Institutes of Health for an accurate biomarker [22]. CgA has been widely regarded as being of no value in the management of lung NEN [23]. The NETest, as shown in other NET studies [11, 16], may provide a more accurate method for assessing treatment response (Supplementary Material, Fig. S3).

ADC and SQC were NETest-positive (score >20) in 42% and 35%, respectively. Although we did not evaluate NETest gene expression in matched blood: tissue samples, the positive NETest scores in NSCLC likely reflect the presence of a neuroendocrine genotype in tumours, as has been previously reported [13, 21]. Whereas the precise biological basis of transcriptomic activation remains unclear, it has been noted that NSCLC that express neuroendocrine mRNA, particularly NETest genes, exhibit aggressive behaviour and have a poor prognosis [13, 24]. Several studies have analysed the relationship between neuroendocrine expression (phenotype and genotype) and prognosis or response to treatment (e.g. chemosensitivity). Although the proportion of NSCLCs with a neuroendocrine phenotype varies depending on the histological technique or marker utilized, transcriptome analyses unequivocally demonstrate that up to a third of all lung neoplasias (non-NETs) exhibit the NETest signature, irrespective of histological analysis [13]. Thus, molecular analyses are likely to be more informative than tissue immunohistochemical analyses in the resolution of the precise genotype of a tumour in evolution. In a different study, using a multiplatform approach, 9 genomic subtypes of NSCLC were distinguished, 3 within SQC and 6 within ADC, with a proportion of ADC and SQC sharing molecular features with NETs [25]. The identification of a neuroendocrine gene expression using blood samples in ADC (42%) and SQC (35%) in our study is consistent with this observation as well as with other gene mapping reports that demonstrate that 30–50% of non-neuroendocrine lung tumours exhibit neuroendocrine gene expression [13, 25]. The identification of neuroendocrine features at the transcript level, which is associated with a poor prognosis [13], conversion to a neuroendocrine phenotype [26] and therapeutic resistance [27] certainly have implications for therapeutic strategies. A blood-based tool may provide a method to facilitate identification of these phenomena in NSCLC (Supplementary Material, Fig. S3).

We noted that 18 (36%) patients with IPF were also NETest-positive. Increasing the cut-off score to 40 reduced this number to 10%. This result probably reflects the well-described phenomenon of neuroendocrine cell hyperplasia and NET neoplasia associated with chronic pulmonary fibrosis [28]. Increasing the NETest cut-off >40 was highly effective in differentiating lung NEN from controls and IPF. Using 20 as a cut-off, the specificity (rule-in a NET diagnosis) was 83% and the PLR was 5.4. At 40, the specificity increased to 97% with a PLR of 17.4. This result indicates that the majority of patients who have a high score (NETest >40) are extremely likely to have a lung NEN (Supplementary Material, Table S7).

Increasing the NETest cut-off score to >40 was also highly effective in separating BPC from ADC and SQC with a specificity of 94% and a PLR of 13.2 versus NSCLC. Thus, whereas an NETest score of >20 identified all tumours with features of NEN, a level of >40 excluded ∼95% of ADC/SQC. NETest scores >40 are indicative of progressive disease in gastroenteropancreatic NET disease [11, 16]. We therefore examined this parameter in the current series and noted that in the BPC group 84% with an NETest score >40 exhibited progressive disease. It seems therefore that elevated levels of the NETest identify an aggressive BPC phenotype. A separate study with further analysis of the omic clusters that constitute the NETest [9] may allow development of a more specific mathematical tool to define the likelihood of tumour progression.

This study, albeit in a heterogeneous group and dependent on local histopathological expertise, indicates that the NETest detected all lung tumours with neuroendocrine elements. In particular, it identified BPC, SCLC and LCNEC with >90% accuracy. Using a cut-off of >40, there is a 95% probability that the lung tumour is ‘neuroendocrine/carcinoid’. In contrast, CgA was non-informative. The use of a liquid biopsy to identify and manage lung tumours with neuroendocrine gene features should be prospectively evaluated.

## Supplementary Material

ezaa018_Supplementary_Data

## Notes

Presented at the 27th European Conference on General Thoracic Surgery, Dublin, Ireland, 9–12 June 2019.

## ABBREVIATIONS

AC: Atypical carcinoid
ADC: Adenocarcinoma
AUC: Area under the curve
BPC: Bronchopulmonary carcinoid
CgA: Chromogranin A
DOR: Diagnostic odds ratio
IPF: Idiopathic pulmonary fibrosis
KW: Kruskal–Wallis
LCNEC: Large-cell neuroendocrine carcinoma
NEN: Neuroendocrine neoplasia
NER: Negative likelihood ratio
NET: Neuroendocrine tumours
NSCLC: Non-small-cell lung cancer
PLR: Positive likelihood ratio
SCLC: Small-cell lung cancer
TC: Typical carcinoid
