# Supplementary material for: Molecular identification of bronchopulmonary neuroendocrine tumours and neuroendocrine genotype in lung neoplasia using the NETest liquid biopsy
Source: Eur J Cardiothorac Surg. 2020 Feb 11;57(6):1195–202. doi: 10.1093/ejcts/ezaa018 (PMC8325497; doi:10.1093/ejcts/ezaa018)
Supplement: ezaa018_Supplementary_Data [file ezaa018_supplementary_data.pdf]

Supplemental Material

Molecular Identification of Bronchopulmonary Neuroendocrine Tumors and Neuroendocrine Genotype in Lung Neoplasia using the NETest Liquid Biopsy

AUTHORS:

Pier Luigi Filosso[1], Kjell Oberg[2*], Anna Malczewska[3], Anna Lewczuk[4], Matteo Roffinella[1], Harry Aslanian[5], Lisa Bodei[6]

AFFILIATIONS:

[1]Department of Surgery, University of Torino, Torino, Italy

[2] Department of Endocrine Oncology, University Hospital, Uppsala, Sweden

[3]Department of Endocrinology, Medical University of Silesia, Katowice, Poland

[4]Endocrinology Unit, Medical University of Gdansk, Gdansk, Poland

[5]Department of Medicine, Yale University School of Medicine, New Haven CT, USA

[6]Department of Radiology, Memorial Sloan Kettering Cancer Center, New York, USA

***CORRESPONDING AUTHOR**:

kjell.oberg@medsci.uu.se

1. Supplemental Figures

Supplemental Figure 1. ROC curve analysis for the biomarkers in the diagnostic cohort (*n*=303).

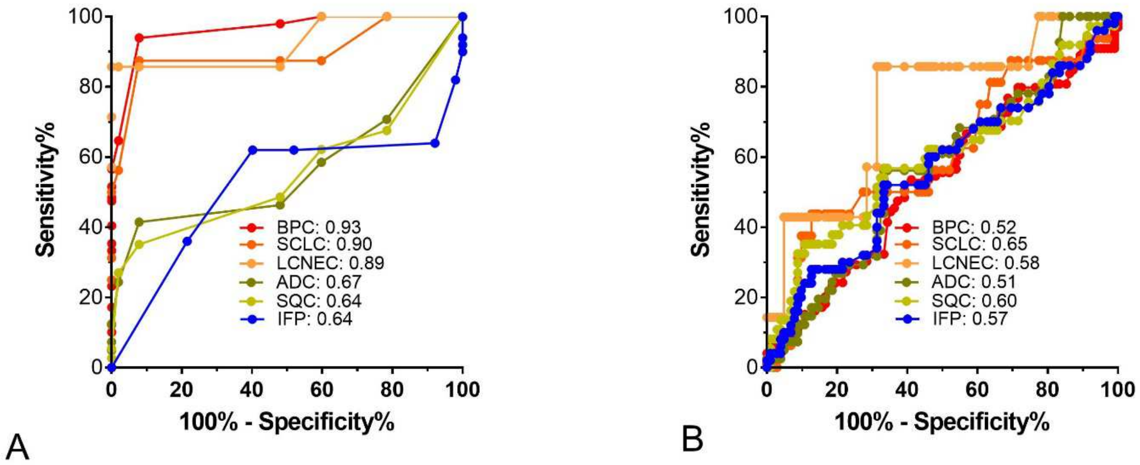

1A. **NETest**: The AUROC for differentiating controls from lung neuroendocrine neoplasia ranged from 0.89 (LCNEC) to 0.93 (BPC) (all $p<0.001$). Non-neuroendocrine neoplasia and benign lung disease (IPF) had AUCs ranging from 0.64 to 0.67 (p=0.75 to 0.22).

1B. **CgA**: The AUROC for differentiating controls from lung neuroendocrine neoplasia ranged from 0.52 (BPC) to 0.65 (SCLC) (p=0.4 to 0.15). Non-neuroendocrine neoplasia and benign lung disease (IPF) had AUCs ranging from 0.51 to 0.60 (p=0.22 to 0.08).

Supplemental Figure 2. Algorithm for lung nodule screening and role of NETest

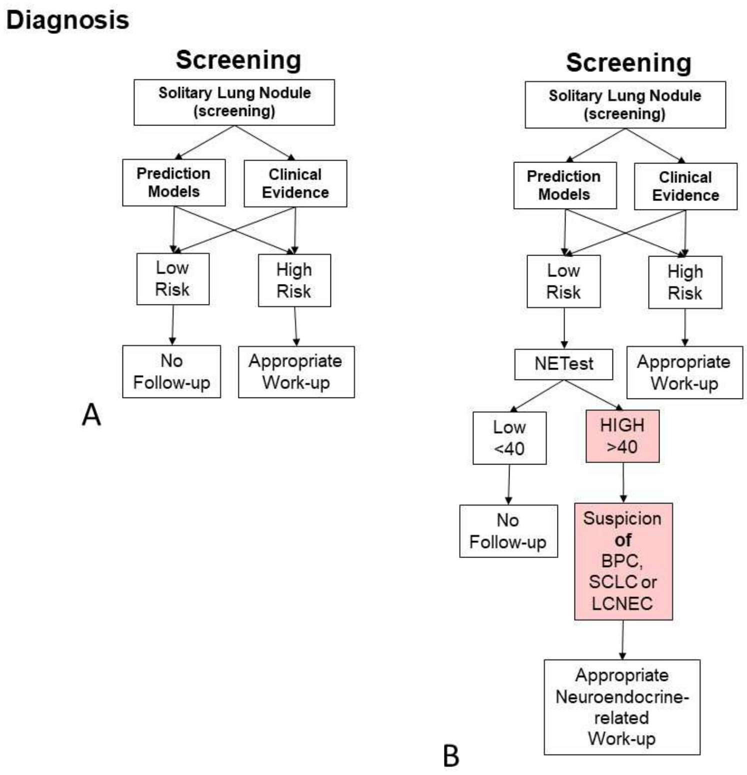

2A. Current diagnostic screening protocols.
Lung nodules that are identified using screening protocols are currently evaluated for risk of disease using a number of techniques. These include using diagnostic algorithms as per the American College of Chest Physicians (ACCP) [1] or the British Thoracic Society (BTS) [2] guidelines which assign patients with lung nodules ≥8 mm in diameter into either a high- or low-risk group. Some guidelines, e.g., BTS, suggest using the Brock University cancer prediction equation for initial risk assessment. This is a logistic regression-based calculator that incorporates various clinical (age, gender, previous history) and imaging parameters (nodule size, appearance). The output is a percentage cancer probability (0-100%). If the risk is high enough (≥10%), a positron emission tomography/CT (PET/CT) scan can be undertaken. The data is then included into Herder model which re-evaluates the risk of malignancy based on inclusion of the PET-CT findings. Typically, these approaches only apply to nodules ≥8mm in size and ultimately result in biopsy for a definitive diagnosis. Smaller nodules and those that show no change in nodule size at follow-up are excluded from follow-up [2]. Clinical judgement is as effective as mathematical models [3], and some guidelines e.g., Fleishner Society [4, 5], suggest a dichotomous risk stratification: low-risk (<5%) group, associated with younger age, less smoking, smaller, smooth, and non-upper lobe nodules and a high-risk (>5%) group, associated with some or all of the opposite

features. Irrespective of the approach, the accuracy of a cancer diagnosis ranges from ~65% (clinical demographics) [6] to 80% (Herder model) [7] and CT/radiomics approaches [8]. In one large recent study comparing the Brock model with the Mayo and VA models [9], while the sensitivities (rule-in) were generally good (24-100%, depending on model threshold) the specificity (rule-in) was typically poor (<50%).

2B. Incorporation of the NETest.

A potential use of the NETest would be in those with nodules considered to have low risk by mathematical calculators or clinical judgement. The specificity of the NETest using a cut-off of 40 is extremely high – 97% – which would provide assurance that a nodule does not express neuroendocrine features. Given that SCLC comprises ~20% of all lung neoplasia and the majority are diagnosed with advanced disease [10], it may be prudent to include the NETest as an additional tool. This is supported by the high positive likelihood ratio for the NETest (17.4) which provides additional assurance that a high score would allow for diagnostic confirmation of neuroendocrine neoplasia. Those that have low NETest scores can therefore follow the current guidelines for low risk, while those with a high score could be appropriately evaluated using neuroendocrine tumor-related protocols e.g., biopsy etc [11].

Supplemental Figure 3. Management algorithm including the NETest

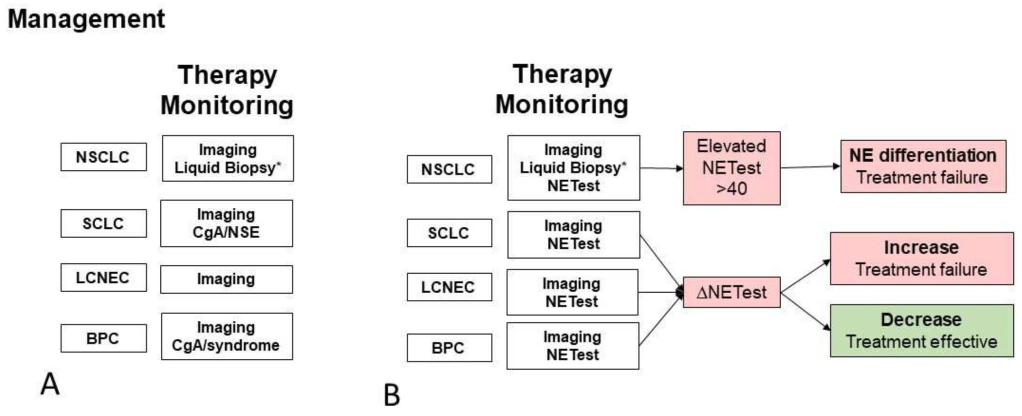

3A. Current management protocols.
Treatment efficacy in non-small cell lung cancers (NSCLC) is currently based on imaging or the use of specific liquid biopsies that are patient-personalize e.g., EGFR mutations [12]. No blood-based assay is used for the detection of conversion to a neuroendocrine phenotype [13] – a key feature of treatment failure [14]. Small cell lung cancers (SCLC) are currently evaluated using imaging and blood biomarkers e.g., chromogranin A (CgA) or NSE (neuron-specific enolase) that have low utility in capturing treatment responsiveness [15, 16]. Imaging is the only modality for large cell neuroendocrine carcinoma (LCNEC) while bronchopulmonary carcinoids (BPC), like SCLC, are currently following using low-efficacy tools [11].
3B. Incorporation of the NETest.
A personalized medicine approach provides a clinically useful approach for monitoring NSCLC with specific mutations. Based on this premise, we would like to suggest the NETest as a tool to help monitoring disease. For individuals with NSCLC, a conversion to an elevated NETest could identify those who may be developing a neuroendocrine genotype/phenotype. In these instances, individuals can undergo biopsy to confirm cell-level changes, which has obvious treatment modification appeal. In neuroendocrine neoplasia, changes in NETest from pre-treatment could be used to identify treatment responsiveness and allow for early intervention. This has previously been determined as appropriate and clinically valuable strategy in gastroenteropancreatic NETs [17-19]; we suggest it may be similarly useful in lung neoplasia.

*These liquid biopsies are typically DNA-related and are measurements of tumor mutations that are detectable in plasma e.g., T790M mutation/COBAS test [20].
ΔNETest = change in NETest from baseline (pre-treatment levels).

2. Supplemental Tables

Supplemental Table 1. Gene panel included in the NETest

Biomarker or Housekeeping Gene		NCBI Chromosome location
Symbol	Name	
AKAP8L	A kinase (PRKA) anchor protein 8-like	Chr.19: 15490859 – 15529833
ALG9	asparagine-linked glycosylation 9, alpha-1,2-mannosyltransferase homolog	Chr. 11 - 111652919 - 111742305
APLP2	amyloid beta (A4) precursor-like protein 2	Chr. 11 - 129939716 - 130014706
ARAF1	v-raf murine sarcoma 3611 viral oncogene homolog	Chr. X - 47420578 - 47431320
ATP6V1H	ATPase, H+ transporting, lysosomal 50/57kDa, V1, Subunit H	Chr.8: 54628115 – 54755850
BNIP3L	BCL2/adenovirus E1B 19kDa interacting protein 3-like	Chr.8: 26240523 – 26270644
BRAF	v-raf murine sarcoma viral oncogene homolog B1	Chr. 7 - 140433812 - 140624564
C21ORF7	chromosome 21 open reading frame 7	Chr.21: 30452873 – 30548204
CD59	CD59 molecule, complement regulatory protein	Chr. 11 - 33724556 - 33758025
COMMD9	COMM domain containing 9	Chr.11: 36293842 – 36310999
CTGF	connective tissue growth factor	Chr. 6 - 132269316 - 132272518
ENPP4	ectonucleotide pyrophosphatase/phosphodiesterase 4	Chr.6: 46097701 – 46114436
FAM131A	family with sequence similarity 131, member A, transcript variant 2	Chr.3: 184053717 – 184064063
FLJ10357	Rho guanine nucleotide exchange factor (GEF) 40 (ARHGEF40)	Chr.14: 21538527 – 21558036
FZD7	frizzled homolog 7 (Drosophila)	Chr. 2 - 202899310 - 202903160
GLT8D1	glycosyltransferase 8 domain containing 1, transcript variant 3	Chr.3: 52728504 – 52740048
HDAC9	histone deacetylase 9, transcript variant 6	Chr.7: 18535369 – 19036993
HSF2	heat shock transcription factor 2, transcript variant 1	Chr.6: 122720696 – 122754264
Ki-67	antigen identified by monoclonal antibody Ki-67	Chr. 10 - 129894923 - 129924655
KRAS	v-Ki-ras2 Kirsten rat sarcoma viral oncogene homolog	Chr. 12 - 25358180 - 25403854
LEO1	Leo1, Paf1/RNA polymerase II complex component homolog (S. cerevisiae)	Chr.15: 52230222 – 52263958
MORF4L2	mortality factor 4 like 2, transcript variant 1	Chr.X: 102930426 – 102943086
NAP1L1	nucleosome assembly protein 1-like 1	Chr. 12 - 76438672 - 76478738
NOL3	nucleolar protein 3 (apoptosis repressor with CARD domain), transcript variant 3	Chr.16: 67204405 – 67209643
NUDT3	nudix (nucleoside diphosphate linked moiety X)-type motif 3	Chr.6: 34255997 – 34360441
OAZ2	ornithine decarboxylase antizyme 2	Chr.15: 64979773 – 64995462
PANK2	pantothenate kinase 2	Chr.20: 3869486 – 3904502
PHF21A	PHD finger protein 21A, transcript variant 1	Chr.11: 45950870 – 46142985
PKD1	polycystic kidney disease 1 (autosomal dominant), transcript variant 2	Chr.16: 2138711 – 2185899
PLD3	phospholipase D family, member 3, transcript variant 1	Chr.19: 40854332 – 40884390
PQB1	polyglutamine binding protein 1, transcript variant 2	Chr.X: 48755195 – 48760422
PNMA2	paraneoplastic antigen MA2	Chr. 8 - 26362196 - 26371483
RAF1	v-raf-1 murine leukemia viral oncogene homolog 1	Chr. 3 - 12625100 - 12705700
RNF41	ring finger protein 41, transcript variant 4	Chr.12: 56598285 – 56615735
RSF1	remodeling and spacing factor 1	Chr.11: 77377274 – 77531880
RTN2	reticulon 2, transcript variant 1	Chr.19: 45988550 – 46000313
SMARCD3	SWI/SNF related, matrix associated, actin dependent regulator of chromatin, subfamily d, member 3, transcript variant 3	Chr.7: 150936059 – 150974231
SPATA7	spermatogenesis associated 7, transcript variant 2	Chr.14: 88851988 – 88904804
SST1	somatostatin receptor 1	Chr.14: 38677204 – 38682268
SST3	somatostatin receptor 3	Chr.22: 37602245 – 37608353
SST4	somatostatin receptor 4	Chr.20: 23016057 – 23017314

SST5	somatostatin receptor 5, transcript variant 1	Chr.16: 1122756 – 1131454
TECPR2	tectonin beta-propeller repeat containing 2, transcript variant 2	Chr.14: 102829300 – 102968818
TPH1	tryptophan hydroxylase 1	Chr. 11 - 18042538 - 18062309
TRMT112	tRNA methyltransferase 11-2 homolog (S. cerevisiae)	Chr.11: 64084163 – 64085033
VMAT1	solute carrier family 18 (vesicular monoamine), member 1	Chr. 8 - 20002366 - 20040717
VMAT2	solute carrier family 18 (vesicular monoamine), member 2	Chr. 10 - 119000716 - 119037095
VPS13C	vacuolar protein sorting 13 homolog C (S. cerevisiae), transcript variant 2B	Chr.15: 62144588 – 62352647
WDFY3	WD repeat and FYVE domain containing 3	Chr.4: 85590690 – 85887544
ZFHX3	zinc finger homeobox 3, transcript variant B	Chr.16: 72816784 – 73092534
ZXDC	zinc finger C, transcript variant 2	Chr.3: 126156444 – 126194762
ZZZ3	zinc finger, ZZ-type containing 3	Chr.1: 78030190 – 78148343

Supplemental Table 2. Diagnostic Metrics for the NETest in different lung pathologies versus controls

Parameters	BPC	SCLC	LCNEC	ADC	SQC	IFP
**Sensitivity**	**94** (87-98)	**88** (62-98)	**86** (42-99)	**41** (26-58)	**35** (20-53)	**36** (23-51)
**Specificity**	**92** (85-97)	**92** (85-97)	**92** (85-97)	**92** (85-97)	**92** (85-97)	**92** (85-97)
**AUC**	**0.93** (0.89-0.96)	**0.90** (0.83-0.95)	**0.89** (0.82-0.94)	**0.67** (0.59-0.75)	**0.64** (0.55-0.72)	**0.64** (0.56-0.72)
**PLR**	**12.0** (6.2-23.5)	**11.2** (5.6-22.3)	**10.9** (5.3-22.7)	**5.3** (2.5-11.3)	**4.5** (2-9.9)	**4.6** (2.1-9.8)
**NLR**	**0.07** (0.03-0.14)	**0.14** (0.04-0.5)	**0.16** (0.02-0.95)	**0.64** (0.49-0.83)	**0.7** (0.55-0.9)	**0.7** (0.56-0.86)
**DOR**	**182** (61-545)	**82** (16-427)	**71** (8-660)	**8.3** (3.2-21.6)	**6.3** (2.4-17.1)	**6.6** (2.6-16.7)
z-statistic	**9.3**	**5.2**	**3.7**	**4.4**	**3.7**	**4.04**
p-value	<0.0001	<0.0001	0.002	<0.0001	0.0002	0.0001

AUC – area under the curve, PLR = positive likelihood ratio, NLR = negative likelihood ratio, DOR = diagnostic odd's ratio

Supplemental Table 3. Diagnostic Metrics for CgA for different lung pathologies versus controls

Parameters	BPC	SCLC	LCNEC	ADC	SQC	IFP
**Sensitivity**	**19** (12-28)	**44** (20-70)	**30** (7-65)	**17** (7-32)	**35** (20-53)	**28** (16-42)
**Specificity**	**85** (72-92)	**85** (72-92)	**85** (72-92)	**85** (72-92)	**85** (72-92)	**85** (72-92)
**AUC**	**0.52** (0.45-0.59)	**0.65** (0.52-0.73)	**0.58** (0.48-0.67)	**0.51** (0.43-0.60)	**0.60** (0.52-0.68)	**0.57** (0.48-0.65)
**PLR**	**1.3** (0.7-2.4)	**3.0** (1.4-6.1)	**2.0** (0.7-5.9)	**1.2** (0.5-2.6)	**2.4** (1.3-4.5)	**1.9** (1.0-3.6)
**NLR**	**0.95** (0.84-1.07)	**0.66** (0.43-1.02)	**0.82** (0.54-1.24)	**0.97** (0.83-1.14)	**0.76** (0.59-0.98)	**0.84** (0.70-1.0)
**DOR**	**1.4** (0.7-2.9)	**4.5** (1.5-14)	**4.4** (0.9-21.4)	**1.2** (0.5-3.2)	**3.1** (1.3-7.5)	**2.3** (1.0-5.2)
z-statistic	0.85	2.6	1.8	0.36	2.6	1.9
p-value	0.4	0.0089	0.07	0.72	0.0099	0.053

AUC – area under the curve, PLR = positive likelihood ratio, NLR = negative likelihood ratio, DOR = diagnostic odd's ratio

Supplemental Table 4. Comparison between the NETest and CgA as a diagnostic for lung pathologies

**Parameters**	**Controls**	**BPC**	**SCLC**	**LCNEC**	**ADC**	**SQC**	**IFP**
**Concordant (N+/C+)**	2	19	6	2	1	7	8
**Concordant (N-/C-)**	81	6	1	0	18	18	26
**Discordant (N-/C+)**	13	0	1	1	6	6	6
**Discordant (N+/C-)**	6	74	8	4	16	6	10
**McNemar *p*-value**	0.17	<0.0001 (Chi^2=72)	0.045 (Chi^2=4.0)	0.37	0.055 (Chi^2=3.7)	0.08	0.45

C = chromogranin A, N = NETest

Supplemental Table 5. Diagnostic Metrics when NETest and CgA results are combined

	**BPC vs ADC**		**BPC vs SQC**	
	**NETest alone**	NETest + CgA	**NETest alone**	NETest + CgA
Accuracy	84.1	82.2	86.2	83.4
Sensitivity	92.6	94.3	92.6	94.3
Specificity	58.4	46.3	64.7	45.7
PPV	86.9	83.9	89.7	85.8
NPV	72.7	73.1	72.7	69.6

NPV = negative predictive value, PPV = positive predictive value

Supplemental Table 6. NETest Diagnostic Metrics for BPC vs. ADC and SQC using a cut-off of 40

Parameter	BPC vs. ADC	BPC vs. SQC
**Sensitivity**	**52** (41-62)	**52** (41-62)
**Specificity**	**93** (80-98)	**95** (82-99)
**AUC**	**0.72** (0.64-0.79)	**0.73** (0.60-0.80)
**PLR**	**7.0** (2.3-21.3)	**9.5** (2.4-37.2)
**NLR**	**0.52** (0.42-0.65)	**0.51** (0.41-0.64)
**DOR**	**12.9** (3.7-44.6)	**17.9** (4.1-78.2)
z-statistic	4.1	3.8
p-value	0.0001	0.0001

AUC – area under the curve, PLR = positive likelihood ratio, NLR = negative likelihood ratio, DOR = diagnostic odd's ratio

Supplemental Table 7. NETest Diagnostic Metrics for lung neuroendocrine neoplasia vs controls and benign disease using cut-offs of 20 and 40

Parameter	NETest: 20	NETest: 40
**Sensitivity**	**93** (87-97)	**54** (48-66)
**Specificity**	**83** (76-89)	**97** (93-99)
**AUC**	**0.88** (0.83-0.91)	**0.77** (0.72-0.82)
**PLR**	**5.4** (3.8-7.7)	**17.4** (7.3-41.9)
**NLR**	**0.09** (0.05-0.17)	**0.44** (0.36-0.54)
**DOR**	**60.8** (27.4-135.4)	**36.6** (15.1-103.5)
z-statistic	10.1	7.5
p-value	0.0001	0.0001

AUC – area under the curve, PLR = positive likelihood ratio, NLR = negative likelihood ratio, DOR = diagnostic odd's ratio

3. References

[1] Gould MK, Donington J, Lynch WR, Mazzone PJ, Midthun DE, Naidich DP *et al. Evaluation of individuals with pulmonary nodules: when is it lung cancer? Diagnosis and management of lung cancer, 3rd ed: American College of Chest Physicians evidence-based clinical practice guidelines*. Chest 2013;**143**:e93S-e120S. doi: 10.1378/chest.12-2351.

[2] Callister ME, Baldwin DR, Akram AR, Barnard S, Cane P, Draffan J *et al. British Thoracic Society guidelines for the investigation and management of pulmonary nodules*. Thorax 2015;**70**:ii1-ii54. doi: 10.1136/thoraxjnl-2015-207168.

[3] Balekian AA, Silvestri GA, Simkovich SM, Mestaz PJ, Sanders GD, Daniel J *et al. Accuracy of clinicians and models for estimating the probability that a pulmonary nodule is malignant*. Ann Am Thorac Soc 2013;**10**:629-35. doi: 10.1513/AnnalsATS.201305-107OC.

[4] MacMahon H, Naidich DP, Goo JM, Lee KS, Leung ANC, Mayo JR *et al. Guidelines for Management of Incidental Pulmonary Nodules Detected on CT Images: From the Fleischner Society 2017*. Radiology 2017;**284**:228-43. doi: 10.1148/radiol.2017161659. Epub 2017 Feb 23.

[5] Bueno J, Landeras L, Chung JH. *Updated Fleischner Society Guidelines for Managing Incidental Pulmonary Nodules: Common Questions and Challenging Scenarios*. Radiographics 2018;**38**:1337-50. doi: 10.148/rg.2018180017.

[6] Wang J, Gao R, Huo Y, Bao S, Xiong Y, Antic SL *et al. Lung Cancer Detection using Co-learning from Chest CT Images and Clinical Demographics*. Proc SPIE Int Soc Opt Eng 2019;**10949.**:10.1117/12.2512965.

[7] Perandini S, Soardi GA, Larici AR, Del Ciello A, Rizzardi G, Solazzo A *et al. Multicenter external validation of two malignancy risk prediction models in patients undergoing 18F-FDG-PET for solitary pulmonary nodule evaluation*. Eur Radiol 2017;**27**:2042-46. doi: 10.1007/s00330-016-4580-3. Epub 2016 Sep 15.

[8] Hawkins S, Wang H, Liu Y, Garcia A, Stringfield O, Krewer H *et al. Predicting Malignant Nodules from Screening CT Scans*. J Thorac Oncol 2016;**11**:2120-28. doi: 10.1016/j.jtho.2016.07.002. Epub 16 Jul 13.

[9] Nair VS, Sundaram V, Desai M, Gould MK. *Accuracy of Models to Identify Lung Nodule Cancer Risk in the National Lung Screening Trial*. Am J Respir Crit Care Med 2018;**197**:1220-23. doi: 10.164/rccm.201708-1632LE.

[10] Travis WD, Brambilla E, Nicholson AG, Yatabe Y, Austin JH, Beasley MB *et al. The 2015 World Health Organization Classification of Lung Tumors: Impact of Genetic, Clinical and Radiologic Advances Since the 2004 Classification*. J Thorac Oncol 2015;**10**:1243-60. doi: 10.097/JTO.0000000000000630.

[11] Caplin ME, Baudin E, Ferolla P, Filosso P, Garcia-Yuste M, Lim E *et al. Pulmonary neuroendocrine (carcinoid) tumors: European Neuroendocrine Tumor Society expert consensus and recommendations for best practice for typical and atypical pulmonary carcinoids*. Ann Oncol 2015;**26**:1604-20. doi: 10.093/annonc/mdv041. Epub 2015 Feb 2.

[12] Lee JY, Qing X, Xiumin W, Yali B, Chi S, Bak SH *et al. Longitudinal monitoring of EGFR mutations in plasma predicts outcomes of NSCLC patients treated with EGFR TKIs: Korean Lung Cancer Consortium (KLCC-12-02)*. Oncotarget 2016;**7**:6984-93. doi: 10.18632/oncotarget.6874.

[13] Park JW, Lee JK, Sheu KM, Wang L, Balanis NG, Nguyen K *et al. Reprogramming normal human epithelial tissues to a common, lethal neuroendocrine cancer lineage*. Science 2018;**362**:91-95. doi: 10.1126/science.aat5749.

[14] Sequist LV, Waltman BA, Dias-Santagata D, Digumarthy S, Turke AB, Fidias P *et al. Genotypic and histological evolution of lung cancers acquiring resistance to EGFR inhibitors*. Sci Transl Med 2011;**3**:75ra26. doi: 10.1126/scitranslmed.3002003.

[15] Isgro MA, Bottoni P, Scatena R. *Neuron-Specific Enolase as a Biomarker: Biochemical and Clinical Aspects*. Adv Exp Med Biol 2015;**867:125-43.**:10.1007/978-94-017-7215-0_9.

[16] Oberg K, Modlin I, DeHerder W, Pavel M, Klimstra D, Frilling A *et al. Biomarkers for Neuroendocrine Tumor Disease: A Delphic Consensus assessment of Multianalytes, Genomics, Circulating Cells and Monoanalytes*. Lancet Oncol 2015;**16**:e435046.
[17] Cwikla JB, Bodei L, Kolasinska-Cwikla A, Sankowski A, Modlin IM, Kidd M. *Circulating transcript analysis (NETest) in GEP-NETs treated with Somatostatin Analogs defines Therapy*. J Clin Endocrinol Metab 2015;**100**:E1437-45.
[18] Pavel M, Jann H, Prasad V, Drozdov I, Modlin IM, Kidd M. *NET Blood Transcript Analysis defines the Crossing of the Clinical Rubicon: When Stable Disease becomes Progressive*. Neuroendocrinology 2017;**104**:170-82.
[19] Bodei L, Kidd M, Modlin IM, Severi S, Drozdov I, Nicolini S *et al. Measurement of circulating transcripts and gene cluster analysis predicts and defines therapeutic efficacy of peptide receptor radionuclide therapy (PRRT) in neuroendocrine tumors*. Eur J Nucl Med Mol Imaging 2016;**43**:839-51. doi: 10.1007/s00259-015-3250-z. Epub 2015 Nov 23.
[20] Odogwu L, Mathieu L, Goldberg KB, Blumenthal GM, Larkins E, Fiero MH *et al. FDA Benefit-Risk Assessment of Osimertinib for the Treatment of Metastatic Non-Small Cell Lung Cancer Harboring Epidermal Growth Factor Receptor T790M Mutation*. Oncologist 2018;**23**:353-59. doi: 10.1634/theoncologist.2017-0425. Epub 2017 Dec 14.
